# Effects of solvent—solvent fractionation on the total terpenoid content and in vitro anti‐inflammatory activity of *Serevenia buxifolia* bark extract

**DOI:** 10.1002/fsn3.2149

**Published:** 2021-01-27

**Authors:** Dieu‐Hien Truong, Nhat Thuy Anh Ta, Thanh Vy Pham, Tan Dat Huynh, Quoc Truong Giang Do, Nguyen Chau Giang Dinh, Cong Danh Dang, Thi Kim Chi Nguyen, Anh Vo Bui

**Affiliations:** ^1^ Faculty of Applied Sciences Ton Duc Thang University Ho Chi Minh City Vietnam

**Keywords:** in vitro anti‐inflammatory, *Serevenia buxifolia* extract, solvent–solvent fractionation, total terpenoid content

## Abstract

*Severinia buxifolia* (Rutaceae) is often used as a traditional medical plant. The present study was carried out to estimate the effects of solvents (petroleum ether and hexane: ethyl acetate) used in liquid–liquid extraction to total terpenoid content (TTC) and in vitro anti‐inflammatory activity of the extracts obtained from *S. buxifolia* bark. The results showed that solvent fractionation increased the TTC compared with crude extracts. The hexane: ethyl acetate bark extract fraction (HEF) had the highest TTC (731.48 µg/ml) in comparison with the petroleum ether bark extract fraction (PEF) (564.81 µg/ml) and the crude extract (CE) (184.26 µg/ml). In addition, one of composition of terpenoid of *S. buxifolia*, namely ursolic acid, was determined by HPLC method from the crude CE and the fractions PEF and HEF: 2.44 μg/g DW, 3.56 μg/g DW and 5.04 μg/g DW, respectively. The samples had an in vitro anti‐inflammatory activity comparable with that of two reference standards (aspirin and indomethacin). Particularly, the HEF fraction had the highest in vitro anti‐inflammatory activity (i.e., albumin denaturation: IC_50_ = 147.91 μg/mL, heat‐induced hemolysis: IC_50_ = 159.91 μg/mL, proteinase inhibition: IC_50_ = 117.72 μg/mL, and lipoxygenase activity: IC_50_ = 90.45 μg/mL). Besides, the preliminary experiments of this study were conducted to determine the influences of maceration factors (solvent type, temperature, and time) for *S. buxifolia* bark extract. The TTC ranged from 453.70 to 842.59 mg linalool/g DW, and the extraction yield from 2.40% to 5.120% in all extracts. Based on TTC and EY, the hexane: acetone mixture is recommended as the optimal solvent to obtain the crude bark extract (CE) at 46°C for 24 hr of maceration. Extracts of *S. buxifolia* bark are a promising source for the treatment of inflammatory diseases.

## Graphical abstract

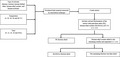

## 1 INTRODUCTION

Among the 20 species of the genus *Atalantia* (within the family Rutaceae) (Roskov et al., 2016), *Severinia buxifolia* (Rutaceae) or *Atalantia buxifolia* is one of the plants of most interest (Safaa et al., 2018). It is frequently used in traditional medicine for the treatment of cough, snakebites, malaria, chronic rheumatism, influenza, and pain (Chang et al., 2018; Truong et al., 2019; Yang et al., 2012). The health benefits of this species are associated with phytochemical components that play a role in human physiology (Wu et al., 2001). Terpenoids (tetraterpenoids and sesquiterpenoids), acridones, and coumarins are the most extensively studied phytochemicals from the branches and roots of this plant (Chang et al., 2018; Shi et al., 2014; Truong et al., 2019; Wu et al., 2001; Yang et al., 2012). For example, Shi et al. (2014) detected a new triterpenoid with an aoptirucallane skeleton in an ethanolic extract of *A. buxifolia* roots. In our previous study, we isolated the acridone alkaloid from *S. buxifolia* branches (Truong et al., 2019). The terpenoid group, mainly containing the volatile organic compounds (VOCs), was found as the main chemical components from the bark of *S. buxifolia* compositions (Vivaldo et al., 2017; Wu et al., 2001). For example, Wu et al. (2001) detected two new tertranortriterpenoids (7‐isovaleroylcycloseverinolide (1) and 7‐isovaleroylcycloepiatalantin (2)) from the root bark of *S. buxifolia*. Generally, terpenoids are an important class of bioactive phytochemicals, but to our knowledge, respective information is limited, especially regarding the influence of solvent fractionation on the content of the components.

In the food and pharmacy industry, separation techniques such as adsorption column chromatography, liquid–liquid extraction, solid–liquid extraction, membrane filtration, or gel filtration chromatography are often applied to obtain pure natural products from complex extracts (Phan et al., 2020; Zhang et al., 2018). Within these methods, various polarities of solvents were applied in the liquid–liquid technique to extract the phytochemical classes from plants, representing a simple and cost‐effective approach (Gharaati, 2019; Sivanandham, 2015). Generally, the selection of a two‐phase solvent system is based on the polarity of components in plant extracts. For example, Canbay (2017) used chloroform, dichloromethane, n‐hexane, and ethyl acetate to obtain target compounds (focusing on volatile compounds) from rose aromatic water. The results showed that n‐hexane was the least suitable liquid–liquid extraction (LLE) solvent. Different solvents for LLE have also been investigated regarding their influence on terpenoid compounds from plant extracts (Pichersky & Raguso, 2018); these authors showed that dichloromethane was the highest optimum extraction solvent for the LLE of rose water terpenoids.

Inflammation is a protective physiological response of living tissues to injury (Chou, 1997; Nurtamin et al., 2018; Rajendran et al., 2013). The characteristics of inflammation are heat, swelling, redness, and pain (George, 2006; Prakash, 2017). The inflammatory response includes the induction of cytokine release, the activity of several enzymes (oxygenases, nitric oxide synthase, and peroxidases), and the expression of cellular adhesion molecules (Gomes et al., 2008; Prakash, 2017). Ruiz‐Ruiz et al. (2017) observed that protein denaturation is also considered as a marker of inflammation. The importance of the inflammatory response is the stabilization of the lysosomal membrane (Chippada et al., 2011). Thus, to evaluate in vitro anti‐inflammatory of plant extracts, stabilization of the human red blood cell membrane (RBC) by a hypo tonicity‐induced membrane can be used as one of the parameters. Terpenoids of plant extracts have potent anti‐inflammatory activity (Prakash, 2017). Moreover, studies have suggested that ursolic acid (UA) is used as a promising molecule with anti‐inflammatory, analgesic, and potential anti‐arthritic activity and diabetes (Ahmad et al., 2018; Bacanlı, 2020). Ursolic acid, found in Citrus plants, is one of the important natural pentacyclic triterpenoid carboxylic acids (Ahmad et al., 2018; Mahlo & Eloff, 2014).

Based on total terpenoid content (TTC) determination and in vitro anti‐inflammatory activity (i.e., inhibition of protein (albumin) denaturation, heat‐induced hemolysis, proteinase activity, and LOX assay), as well as investigation of the extracts obtained from *S. buxifolia* bark, we estimated the effects of solvents (petroleum ether and hexane: ethylacetate) used in LLE in comparison with those of the crude bark extract. TLC and HPLC were also applied to detect the presence of natural oleanolic acid (OA) and ursolic acid (UA) in the bark extracts as compared to pure compound (OA and UA, Sigma, Singapore). In addition, in the preliminary experiments, single‐factor extraction was used to optimize the recovery of terpenoid content and extraction yield from *S. buxifolia* bark by studying the influence of four factors, namely solvent type, maceration temperature, and time.

## 2 MATERIALS AND METHODS

### 2.1 Chemicals

Hexane, diethyl ether, petroleum ether, acetone, ethyl acetate, methanol, dimethyl sulfoxide, oleanolic acid (≥97%) and ursolic acid (UR) (≥97%) were purchased from Sigma‐Aldrich, Singapore. TLC F254 plates, aspirin, and indomethacin were purchased from Merck, Singapore. All chemicals and solvents were of analytical grade.

### 2.2 Plant collection and treatment

The bark was collected from *S. buxifolia* trees in Phu Loc district (Thua Thien Hue Province, Vietnam). The plants were taxonomically identified by the Botany Research and Development Group of Vietnam (Vietnam). Bark was obtained by removing the leaves, trunks, and thorns, then washed with distilled water to remove debris, cut into small pieces, and dried at 40°C for 10 days. The dried bark was then ground into powder using a mill (Jehmlich, Germany) and kept in a dry, airtight container for further usage.

### 2.3 Influence of extraction parameters on total terpenoid content and extraction yield

Single‐factor extraction was used to optimize the recovery of terpenoid compounds and extraction yield from *S. buxifolia* bark by studying the influence of four factors, namely extraction solvent, extraction temperature, and maceration time. First, 400 ml of extracting solvent was added to 20 g of *S. buxifolia* bark powder (1:20, w/v) in a 1,000‐ml beaker. The mixture was then macerated in a water bath (LABEC, Marrickville, Australia) at the respective temperature and time. Subsequently, the mixture was homogenized at the respective temperature by constant shaking for 4 hr, using a homogenizer (IKA, Germany). The filtrate was removed from the residue by filtration using Whatman No. 1 filter paper. This process was repeated three times to exhaustively isolate the plant material, and the obtained extracts were combined. The extract solutions were concentrated using a rotary evaporator (Pollab, India) at 40°C and drying at room temperature. Dried extract samples were stored in an airtight container at 4°C. All experiments were performed in triplicate. The yield of *S. buxifolia* dried extract was calculated according to the following equation:

(1) $$\text{Extraction yield (EY) (\textbackslash{}\% )}=\left(W_{1}\times 100\right)/W_{2},$$

where *W*_1_ is the weight of the extract after evaporation of the solvent and *W*_2_ is the dry weight of the bark sample.

#### 2.3.1 Extraction solvent

By fixing maceration temperature (66°C) and time (24 hr), samples were extracted using binary solvents (namely hexane; acetone; hexane: diethyl ether (1:1, v/v); hexane: ethyl acetate (1:1, v/v); and hexane: acetone (1:1, v/v)). The best extraction solvent was selected based on the value of TTC and EY of extracts.

#### 2.3.2 Maceration temperature

By using the optimal extraction solvent as determined in first step, samples were macerated at different temperatures, namely 6, 26, 46, 66, and 86°C by fixing the maceration time constant at 24 hr. The best maceration temperature was selected based on the values of TTC and EY of extracts.

#### 2.3.3 Maceration time

By using the optimal extraction solvent and maceration temperature as determined in first step and second step, respectively, samples were macerated for 0, 12, 24, 36, and 48 hr. The best maceration time was selected according to the values of TTC and EY of extracts.

### 2.4 Fractionation by liquid–liquid extraction

**FIGURE 1:**
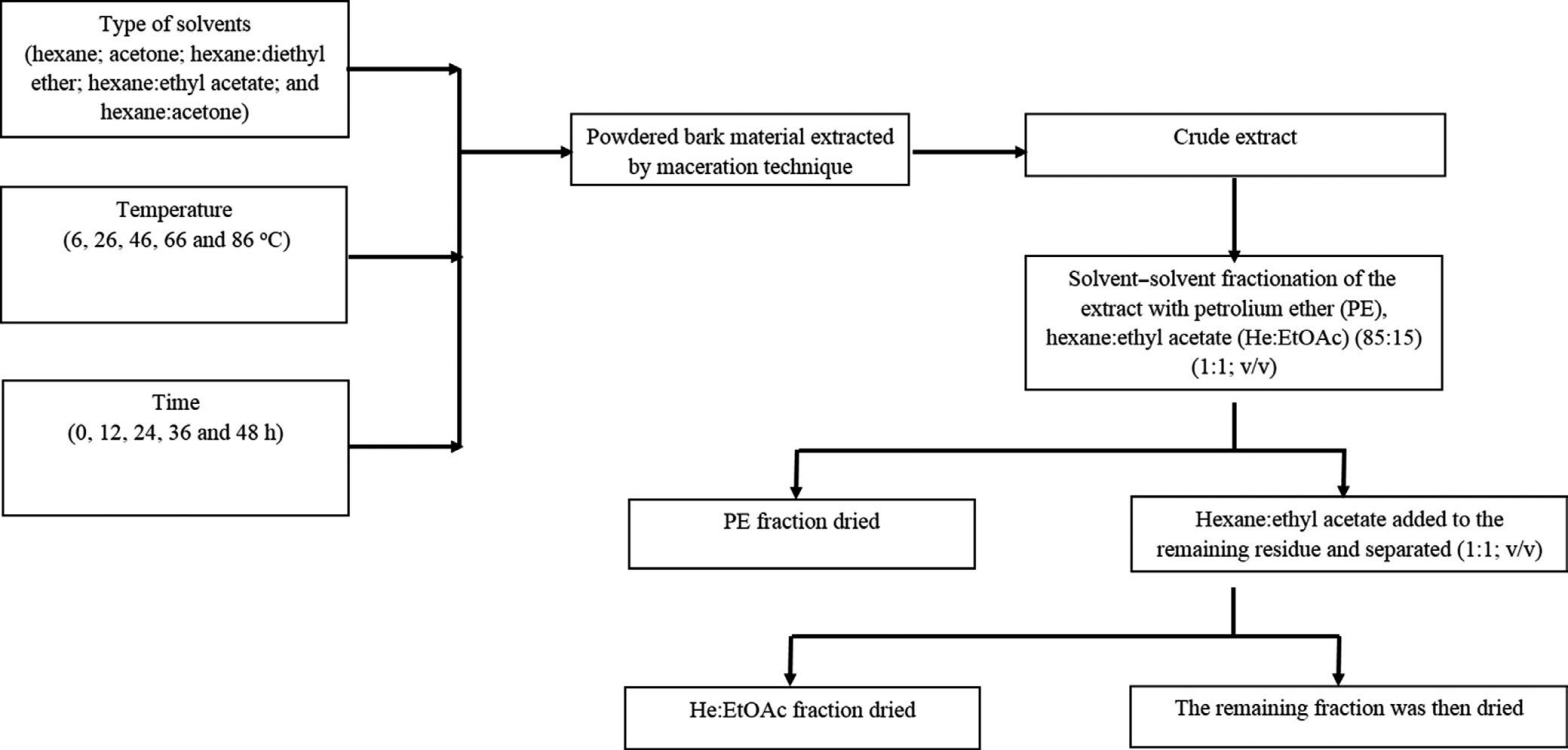
Protocol for the solvent–solvent fractionation of extract components of *Severinia buxifolia* bark

The crude extract of *S. buxifolia* bark was obtained at optimum extracting conditions (solvent, temperature, and time), determined in previous experiments (2.3). The crude bark extract (CE) was then dissolved in steriled distilled water in a separating funnel, equilibrated, and successively extracted with petroleum ether, hexane: ethyl acetate (85:15; v/v) (Sigma‐Aldrich, Singapore) to obtain fractions of various polarities. The fractionation protocol is shown in Figure 1; fractionation was performed in triplicate. The organic fractions (petroleum ether or hexane: ethyl acetate) were concentrated by a rotary evaporator at 40°C, followed by drying at room temperature.

### 2.5 Determination of chemical components by TLC method

The TLC procedure described by Gambhava et al. (2013) was used to determine the number of chemical components in the crude CE, the petroleum ether bark extract fraction (PEF), and the hexane: ethyl acetate bark extract fraction (HEF) of *S. buxifolia*, with some minor modifications. Merck TLC F254 plates were loaded with 30 µl of the extracts, and the prepared plates were then developed in hexane: ethyl acetate: methanol (8.2:1.8:0.5; v/v/v). Oleanolic acid and ursolic acid were considered as commercial standards and prepared by dissolving in absolute methanol (100 µg/ml). The chromatograms were dried at 105°C for 5 min to remove solvents. The chemical components of the extracts were identified by either using UV light (254 and 365 nm) or by spraying with Liebermann reagent (10% H_2_SO_4_ in ethanol and 10% acetic anhydride (Sigma‐Aldrich, Singapore)) solution and then dried at 105°C. Identification of the components of *S. buxifolia* bark extracts was carried out by comparison of the retention factor (Rf) of the various spots. The Rf was obtained by using a meter rule to measure the distance moved by the solvent and the distance moved by the spot; based on this, the retention factor (Rf values) of the various spots was calculated using the following equation:

(2) $$\text{Rf}=S_{1}/S_{2},$$

where *S*_1_ is the distance travelled by the solute and *S*_2_ is the distance travelled by solvent front on the TLC plates.

### 2.6 High‐performance liquid chromatography (HPLC) technique analysis

We used a high‐performance liquid chromatography (HPLC‐2160, Agilent, USA) equipped with a UV detector (Agilent Series 1,100) and an Eclipse Plus C18 column (4.6 × 250 mm) to identify bioactive terpenoid compounds in the extracts of *S. buxifolia* bark. The program was set up as described by Zhang et al. (2013) with some modifications. The mobile phase was composed of acetonitrile (solvent A) and water containing 0.1% phosphoric acid (solvent B): 0–25 min at 22%–23% (solvent A) and 1.0–1.5 ml/min, 25–40 min at 23% (solvent A) and 1.5–1.0 ml/min, and 40–60 min at 23%–90% (solvent A) and 1.0 ml/min. The column temperature was maintained at 25°C; 20 µl of the sample dissolved in MeOH (20–100 ppm) was injected into the column, and detection was attained at 210 nm. Ursolic acid (Sigma, Singapore) at 0.5 mg/ml was applied as the reference standard. The peaks were identified on the basis of retention time (RT) in comparison with the RT of standard ursolic acid. The detected peaks from the extracts were quantified based on peak area.

### 2.7 Determination of total terpenoid content

The total terpenoid content (TTC) of the extracts of *S. buxifolia* bark was determined by the method of Ghorai et al. (2012). To 1 ml of the extracts, we added 2 ml of chloroform. The sample mixture was then vortexed thoroughly before being left for 3 min. Subsequently, 200 μl of concentrated sulfuric acid (H_2_SO_4_) was poured into the mixture, followed by incubation at room temperature for 1.5–2 hr in the dark. A reddish‐brown precipitate was formed in the mixture during incubation. After that, the supernatant was carefully decanted without disturbing the precipitation, and 3 ml of absolute methanol was added and vortexed well until complete dissolving of the precipitation in methanol. Absorbance was read at 538 nm using a visible spectrometer (V‐730 UV‐Vis Spectrophotometer, Jasco, USA). The TTC of the extracts was calculated as mg of linalool per gram of extract (dry weight, DW). The equation of the standard curve was *y* = 0.0036*x* − 0.001, where *R* ^2^ = 0.9927.

### 2.8 Determination of in vitro anti‐inflammatory activity

The in vitro anti‐inflammatory activity of the *S. buxifolia* extracts was determined via assessment of the inhibition of albumin denaturation, the membrane stabilization test (heat‐induced hemolytic and protein inhibitory action), and antilipoxygenase (anti‐LOX) activity, as described by previous studies with minor modifications (Eshwarappa et al., 2016; Govindappa et al., 2011; Leelaprakash & Mohan, 2011; Shaikh et al., 2018; Truong et al., 2019). The extracts of *S. buxifolia* bark (CE, PEF, and HEF) were serially diluted in dimethyl sulfoxide (DMSO) from 25 to 200 μg/ml. Aspirin and indomethacin (Sigma‐Aldrich, Singapore), reference standard anti‐inflammatory drugs, were used as positive controls.

#### 2.8.1 Inhibition of albumin denaturation

We followed the methods of Govindappa et al. (2011), with slight modifications. The reaction mixture consisted of the test extract (1 ml) and 1% aqueous solution of bovine albumin fraction (1 ml). The pH of the reaction mixture was adjusted to 6.3 using 0.1 N HCl at 37°C. The sample extracts were incubated at 37°C for 20 min and then heated to 51°C for 20 min. After cooling to room temperature, turbidity was measured spectrophotometrically at 660 nm. The DMSO was used as control. Percent inhibition of albumin denaturation was calculated as follows:

(3) $$\%\;\text{inhibition of albumin}=\left[\left(A1‐\;A2\right)/A1\right]\;\times 100,$$

where *A*1 = absorption of the control and *A*2 = absorption of the test sample mixture.

#### 2.8.2 Membrane lysis assay

##### Preparation of red blood cell (RBC) suspension

The RBC suspension was prepared according to the method described in Gunathilake et al. (2018) with some modifications. The blood sample was collected from a healthy human volunteer who had not used any nonsteroidal anti‐inflammatory drugs for 2 weeks prior to the experiment. The blood cells were centrifuged at 1008 *g* for 10 min in heparinized centrifuge tubes and washed with an equal volume of normal saline (0.9% NaCl) (three times). After centrifugation, the volume of blood was measured and reconstituted as a 10% (v/v) suspension with normal saline.

##### Heat‐induced hemolysis

This test was carried out as described by Gunathilake et al. (2018), with some modifications as described in Truong et al. (2019). Briefly, 1 ml of blood cell suspension was mixed with 1 ml of test extract of *S. buxifolia* bark; instead of the test sample, only saline was added to the control test tube. All centrifuge tubes containing reaction mixture (2 ml) were incubated in a shaking water bath at 56°C for 30 min. After incubation, the mixture was cooled down rapidly to room temperature and centrifuged at 700 *g* for 5 min to obtain the supernatant. The absorbance of the supernatant was measured at 560 nm using a spectrophotometer (V‐730 UV‐Vis Spectrophotometer, Jasco, USA). The level of hemolysis was calculated using the following equation:

(4) $$\%\;\text{inhibition of hemolysis}=\left[\left(A1‐A2\right)/A1\right]\times 100,$$

where *A*1 = absorption of the control and *A*2 = absorption of the test sample mixture.

##### Proteinase inhibitory activity

Proteinase inhibitory activity of the *S. buxifolia* bark extracts was determined according to the method of Leelaprakash and Mohan (2011), modified by Truong et al. (2019). Briefly, the reaction solution consisting of 1 ml of 20 mM Tris‐HCl buffer (pH 7.4) and 0.06 mg of trypsin was mixed with 1 ml of the tested extract. The mixture was then incubated (37°C for 5 min) before adding 1 ml of 0.7% (w/v) casein, followed by further incubation for an additional 20 min. Subsequently, 1 ml of 70% perchloric acid (HClO_4_) was added to terminate the reaction. The reaction mixture was then centrifuged at 4°C (3,000 rpm, 10 min), and the absorbance of the supernatant was measured at 210 nm against buffer as the blank. As control, phosphate‐buffered solution was used. The percentage inhibition of protein denaturation was calculated using the following equation:

(5) $$\%\;\text{inhibition of denaturation}=\left[\left(A1‐A2\right)/A1\right]\times 100,$$

where *A*1 = absorption of the control and *A*2 = absorption of the test sample mixture.

##### Lipoxygenase (LOX) inhibition assay

Lipoxygenase (LOX) inhibition activity of the extracts of *S. buxifolia* bark was assayed according to the method of Eshwarappa et al. (2016), with some minor modifications. Briefly, we used linoleic acid as substrate and lipoxidase as enzyme, both purchased from Sigma, Singapore. A mixture of a solution of sodium borate buffer (0.8 ml, 0.1 M, pH 8.8) and lipoxygenase (0.08 ml, final concentration 20,000 U/ml) was incubated with 0.8 ml of the tested extract in a 2‐mL cuvette for 5 min at 25°C. After incubation, 0.08 ml of linoleic acid solution (0.6 mM) was added and mixed well, and absorbance was measured at 234 nm. Indomethacin was used as reference standard, whereas phosphate‐buffered solution was used as the control; the percentage inhibition of lipoxygenase was calculated using the following equation:

(6) $$\%\;\text{inhibition of LOX}=\left[\left(A1‐A2\right)/A1\right]\times 100,$$

where *A*1 = absorption of the control and *A*2 = absorption of the test sample mixture.

The results of in vitro anti‐inflammatory activity of *S. buxifolia* extracts were also reported as IC_50_ values. The IC_50_ is defined as the concentration sufficient to obtain 50% of a maximum scavenging capacity. All tests and analyses were run in triplicate, and the obtained values were averaged.

### 2.9 Statistical analysis

All experiments were performed in triplicate, and the results are given as the mean ± standard deviation (±*SD*) for total terpenoid content as well as all in vitro assay tested. Statistical comparisons were carried out by analysis of variance (ANOVA) using the Minitab 15 software and Tukey's multiple comparison test; *p* values < .05 were considered significant.

To reveal the patterns of variation and clustering among treatments, the obtained data were analyzed by means of multivariate analysis, employing hierarchical cluster analysis (HCA), and principal components analysis (PCA). Square Euclidean distances were used to calculate the distances between tested extracts of *S. buxifolia* bark. Each calculated principal component was validated using “full cross‐validation,” with 95% confidence level on parameters. For HCA, the method used was complete linkage. The dendrogram similarity scales generated by the Minitab program ranged from zero (greater similarity) to 5.64 (lower similarity). For PCA, transformed values of variables (average zero and standard deviation 1), called Z scores, were used.

## 3 RESULTS

### 3.1 Effect of extraction conditions (solvent, temperature, and duration time of maceration) on total terpenoid content (TTC) and extraction yield (EY) of *Severinia buxifolia* bark

#### 3.1.1 Extraction conditions–variable interaction through hierarchical clustering and PCA analysis

**FIGURE 2:**
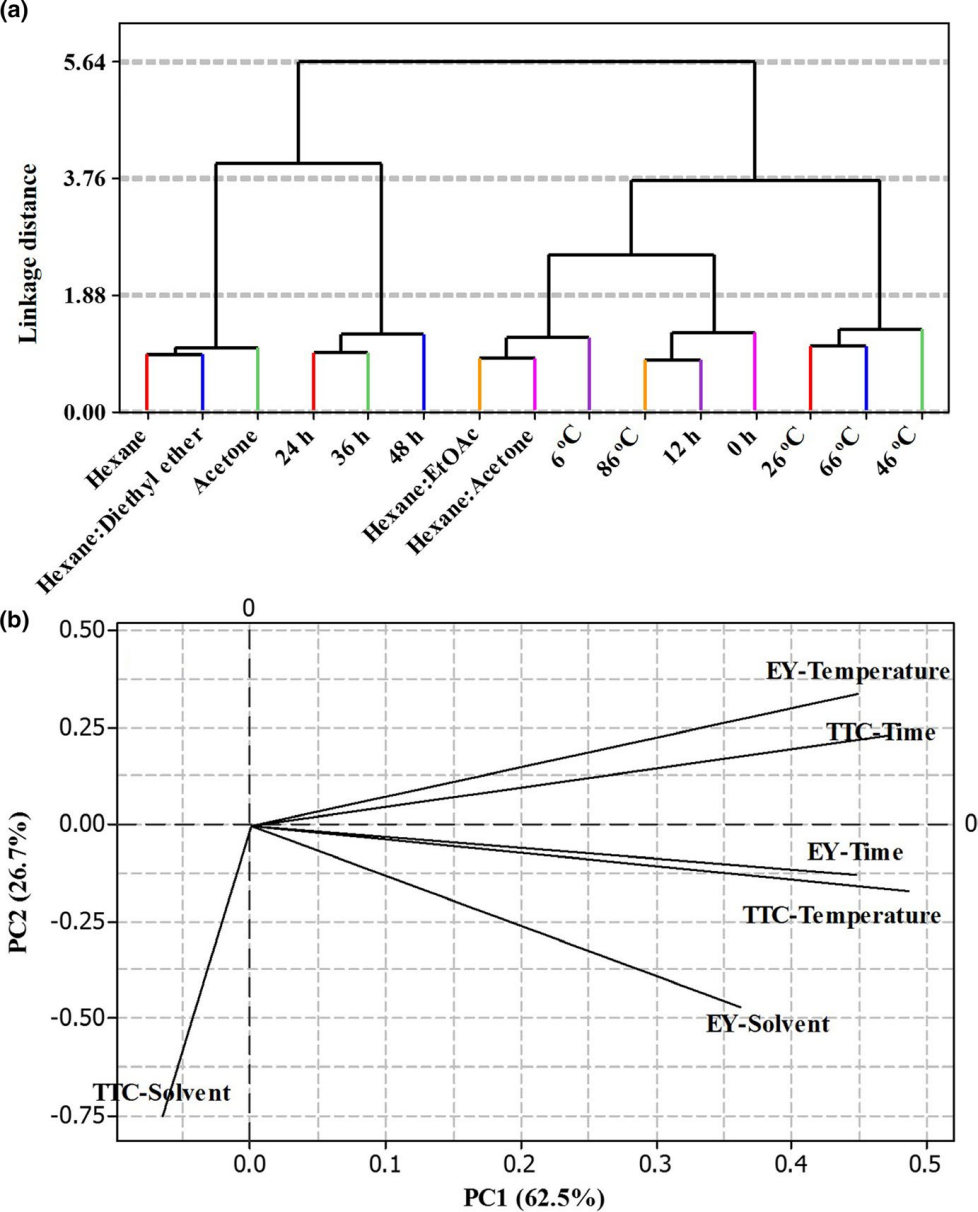
Dendrogram obtained by hierarchical cluster analysis (a) and loading plot of principal component analysis (PCA) (b) using means of total terpenoid content and extraction yield of *Severinia buxifolia* bark over different extracting conditions (temperature, time, and solvent type of maceration)

Hierarchical clustering and principal components analysis (HCA and PCA) were performed to distinguish the relationship between the different factors of the maceration technique (solvents, temperature, and time) on the basis of the TTC and EY of the *S. buxifolia* extracts (Figure 2). The content of total terpenoids varied from 453.70 to 842.59 mg linalool/g DW, and the yield of *S. buxifolia* extracts ranged from 2.40% to 5.10%. The dendrogram (Figure 2a) of the HCA shows the existence of five groups. Examination of the data clearly indicated variations between cluster 1 and cluster 5. This corresponded to treatments with some solvents (cluster 1: hexane, hexane: diethyl ether, and acetone) and to the maceration temperature (cluster 5:26, 46, and 66°C). Cluster 2 represented the effects of increasing maceration time (from 24 to 48 hr) on the TTC and EY of *S. buxifolia* extracts, whereas the bark extracted at the highest temperature (86°C) and at shorter maceration times (0 and 12 hr) was distinguished in cluster 4. The treatments with the lowest temperature (6°C) and two other solvents (hexane: EtOAc and hexane: acetone) belonged to cluster 3.

The PCA using the data set up the mean of TTC and EY of the *S. buxifolia* extracts over three factors of maceration technique (solvent, temperature, and time) (Figure 2b). Followed by HCA, analysis of the score plots constructed with PC1 and PC2 revealed that the TTC and EY of *S. buxifolia* extracts differed among various maceration factors. The first two principal components (PCs) explained 89.2% of the observed variation, that is, PC1 62.5% and PC2 26.7%. The correlations between variables on the first two principal components showed that PC1 was positively correlated with most of factors, that is, TTC‐Temperature (0.487), EY‐Temperature (0.450), TTC‐Time (0.472), EY‐Time (0.448), and EY‐Solvent (0.362), but negatively correlated with only TTC‐Solvent (−0.065). The PC2 was positively correlated with EY‐Temperature (0.341) and TTC‐Time (0.231) and negatively correlated with TTC‐Temperature (−0.172), EY‐Time (−0.129), TTC‐Solvent (−0.750), and EY‐Solvent (−0.470).

#### 3.1.2 Effect of extraction solvent on TTC and EY of *Severinia buxifolia* bark

**FIGURE 3:**
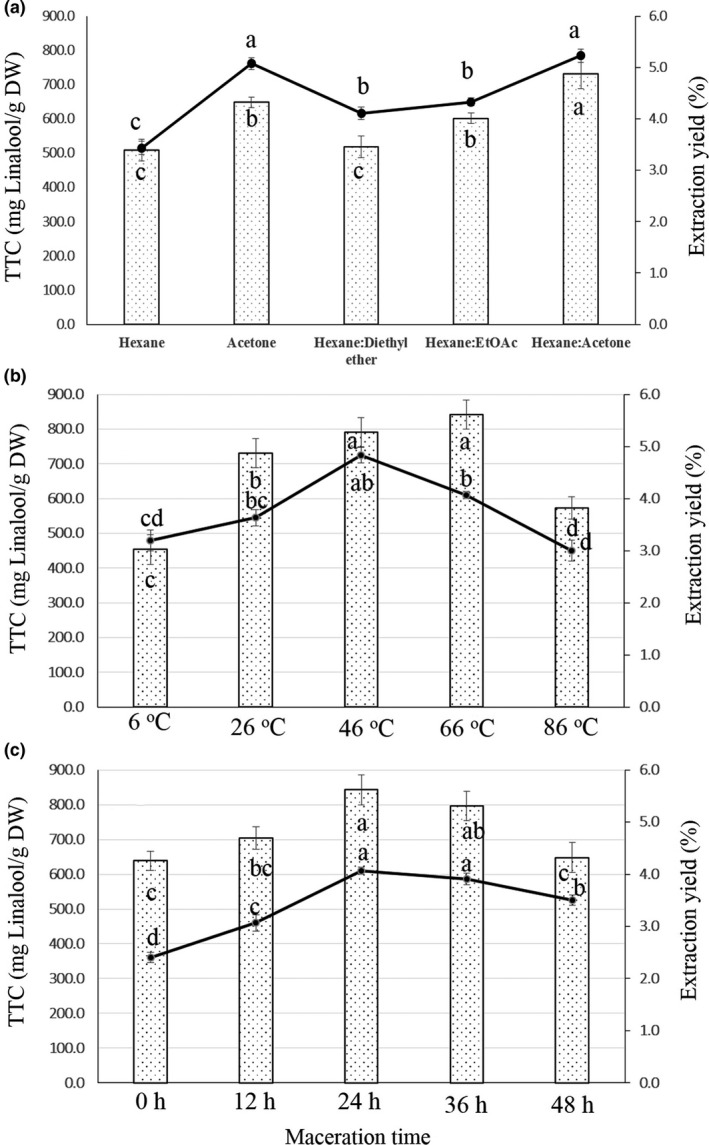
Yield of extraction (line) and total terpenoid content (dot bar) from *Severinia buxifolia* extracts over different factors of maceration: solvent type (a); temperature (b); and time (c). TTC of all samples was measured by colorimetric method with Linalool as the standard reagent. Means within line or bars with different letters significantly differ by Tukey's test at *p* < .05

Based on the ANOVA results, there were significant differences between bark extracts (*p* < .001) (Figure 3a). Different solvents (hexane, acetone, hexane: diethyl ether, hexane: ethyl acetate, and hexane: acetone) were used to extract phytochemicals from the bark of *S. buxifolia*, and the results indicated that the extraction solvents had different impacts on *S. buxifolia* extracts (*p* < .001) regarding TTC and EY values. Concerning the TTC of *S. buxifolia* extracts, the terpenoid assay showed that TTC significantly varied based on the solvents used for extracting chemical components from *S. buxifolia* bark (*p* < .001). The TTC values ranged from 509.26 mg linalool/g DW for hexane extract to 731.48 mg linalool/g DW for hexane: acetone extract. The TTC of the hexane: diethyl ether extract (518.51 mg linalool/g DW) was not significantly higher than that of the hexane extract; conversely, the TTC of the acetone extract was significantly higher (648.15 mg linalool/g DW) than that of the hexane extract. Also, the TTC of the hexane: EtOAc extract was not significantly lower (601.85 mg linalool/g DW) than that of the acetone extract. Crude extract yield ranged from 3.43% for hexane extract to 5.23% for hexan: acetone extract (Figure 3a). The yield of the acetone extract (5.08%) was only slightly lower than that of the hexane: acetone extract, whereas the yield of hexane: diethyl ether extract (4.11%) and of the hexane: ethyl acetate extract (4.33%) were significantly higher than that of the hexane extract. Based on the results of TTC and EY, the best extracting solvent for *S. buxifolia* bark was hexane: acetone (1:1, v/v).

#### 3.1.3 Effect of maceration temperature on TTC and EY of *Severinia buxifolia* bark

The influences of maceration temperature (from 6 to 86°C) on the TTC and EY values of *S. buxifolia* extracts are shown in Figure 3b. The TTC ranged from 453.70 mg linalool/g DW for the bark extract at 6°C to 842.59 mg linalool/g DW for the extract at 66°C. The TTC of *S.buxifolia* extract at 46°C (815.52 mg linalool/g DW) was not significantly lower than that of the extract at 66°C. Conversely, the TTC of the bark extract at 26°C (731.48 mg linalool/g DW) was significantly higher than that of the extract at 6°C (453.70 mg linalool/g DW). Extract yield ranged from 3.00% for the bark extract at 86°C to 4.83% for the extract at 46°C. Yield at 66°C (4.07%) was significantly lower than that at 46°C. Both parameters had no significant influence on the extraction yield at 6°C (3.20%) and 26°C (3.63%). Generally, based on the TTC and EY results, the most suitable maceration temperature for extracting *S. buxifolia* bark is 46°C.

#### 3.1.4 Effect of maceration time on TTC and EY of *Severinia buxifolia* bark

The TTC and EY values varied significantly for different maceration times (*p* < .001) (Figure 3c). The TTC ranged from 638.89 mg linalool/g DW for the *S. buxifolia* extract with a maceration time of 0 hr to 842.59 mg linalool/g DW for the bark extract with a maceration time of 24 hr. The TTC of the extract for 36 hr (790.36 mg linalool/g DW) was not significantly lower than that of the extract for 24 hr. Both parameters had no significant influence on the bark extract with a maceration time of 12 hr (703.70 mg linalool/g DW) and 48 hr. Based on the results for TTC and EY, the optimal maceration time for the extraction of *S. buxifolia* bark is 24 hr.

### 3.2 *Fractionation by liquid*–*liquid extraction*

Based on the results for TTC and EY of the preliminary experiments (3.1), the mixture solvent (hexane: acetone (1:1, v/v) was used to obtain the crude extract of *S. buxifolia* bark at 46°C for 24 hr of maceration. Organic solvents such as petroleum ether and a mixture of hexane: ethyl acetate (85:15, v/v) were used to partition the crude extract via liquid–liquid extraction.

#### 3.2.1 Multivariate data analyses separated *Severinia buxifolia* bark extracts of liquid–liquid extraction

**FIGURE 4:**
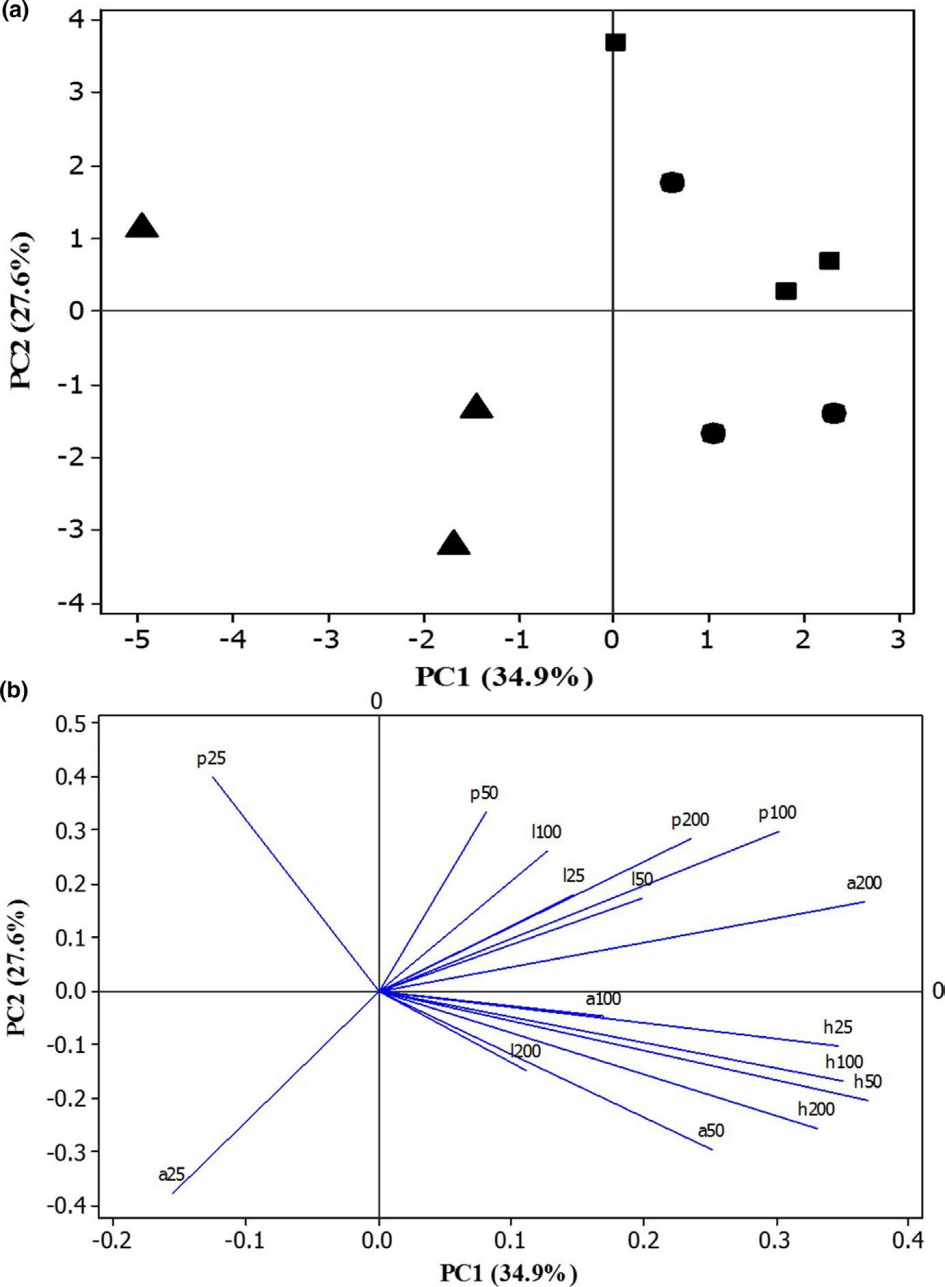
Scores (a) and Loading (b) plots of principal component analysis (PCA) calculated using means of total terpenoid content (TTC) and percentage of inhibition (%) of in vitro anti‐inflammatory activity (inhibition of albumin denaturation, heat‐induced hemolysis, proteinase activity, and lipoxygenase (LOX) assay). Symbols of Scores (a): ●: crude bark extract (CE); ■: hexane:ethyl acetate bark extract fraction (HEF); and ▲: petroleum ether bark extract fraction (PEF)

Based on the results of the PCA, there was a clear pattern of the influence of fraction solvents on the in vitro anti‐inflammatory capacities of *S. buxifolia* bark extracts (Figure 4). The four inhibition assays (albumin denaturation, heat‐induced hemolysis, proteinase activity, and lipoxygenase assay) for crude extract (CE) and fractions (PEF and HEF) at different concentrations (from 25 to 200 μg/mL) were determined and analyzed via PCA (Figure 4). The first two components accounted for 62.5% of the observed variation (PC1 34.9% and PC2 27.6%). Figure 4a indicated that the petroleum ether fraction at different concentrations was highly separated in PC1 of PCA (left side of the score plot); the other fractions and crude extracts were loaded at the opposite side (positive correlation). The PC2 was positively correlated with one petroleum ether concentration, all hexane: ethyl acetate fraction concentrations, and one crude extract concentration, while it was negatively correlated with two concentrations of petroleum ether and crude extract. The first principal component (PC1) separated the inhibition of albumin and proteinase of extracts at 25 μg/mL (left side of the loading plot in Figure 4b) from the extract and the fractions; the other inhibitions at the different concentrations of the extracts were loaded on the opposite side (positive correlation) of the loading plot (Figure 4b). The second principal component (PC2) separated the in vitro anti‐inflammatory assays of albumin denaturation (from 25 to 100 μg/mL of extract concentrations), heat‐induced hemolysis (from 25 to 200 μg/mL of extract concentrations), and lipoxygenase inhibition at the highest extract concentration (200 μg/mL) (below loading plot in Figure 4b). Conversely, the other inhibitions at various concentrations of fractions and crude extracts were loaded on the opposite side (positive correlation) of the loading plot (Figure 4b). Overall, analysis of the score and loading plots constructed with PC1 and PC2 revealed the in vitro anti‐inflammatory activity responsible for differences among different tested extracts at various concentrations (Figure 4).

#### 3.2.2 Changes in the TTC of *Severinia buxifolia* bark extracts

**FIGURE 5:**
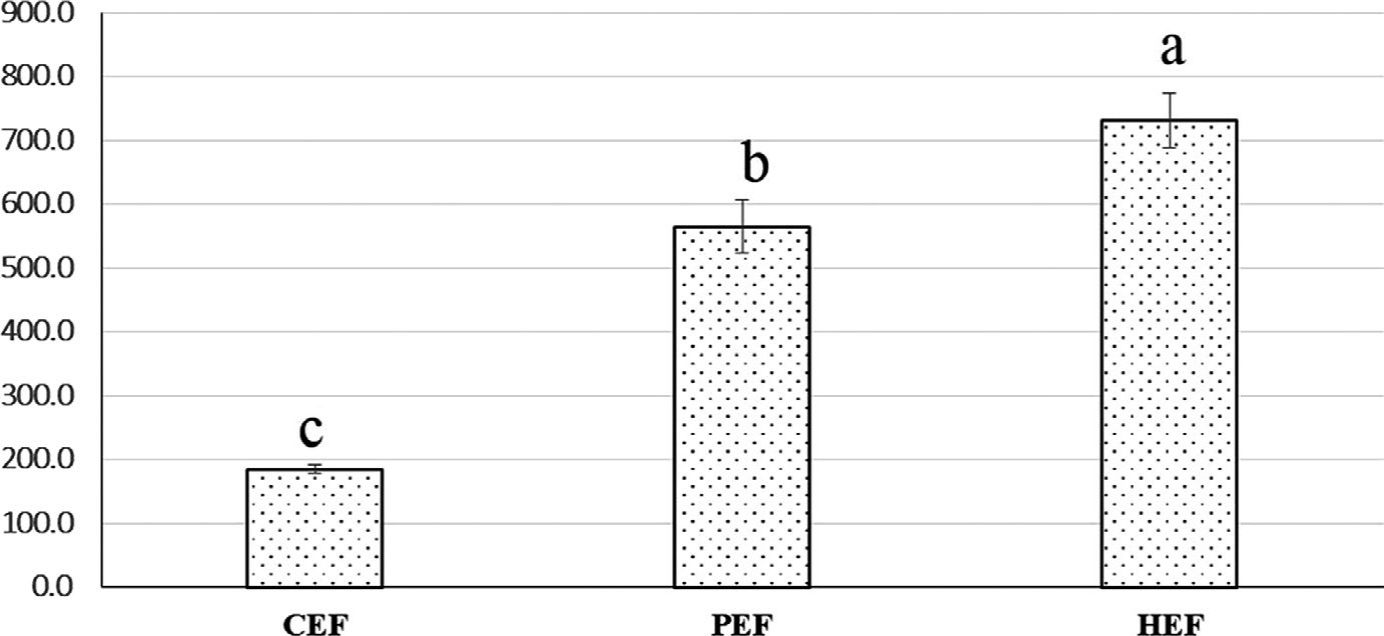
Total terpenoid content (dot bar) of the crude bark extract (CE) and the bark extract fractions (petroleum ether (PEF) and hexane:ethyl acetate (HEF)) obtained by liquid–liquid extraction. Means within bars with different letters significantly differ by Tukey's test at *p* < .05

The TTC levels of crude extract and fractions are presented in Figure 5. The TTC value significantly increased from the crude extract to the fractions (*p* < .001). The highest value was found for the HEF fraction (731.48 mg linalool/g DW), whereas the crude CE had the lowest TTC value (184.26 mg linalool/g DW). The TTC value of PEF was significantly higher than that of the crude CE (564.81 mg linalool/g DW) (*p* < .001).

#### 3.2.3 TLC and HPLC profiling

**FIGURE 6:**
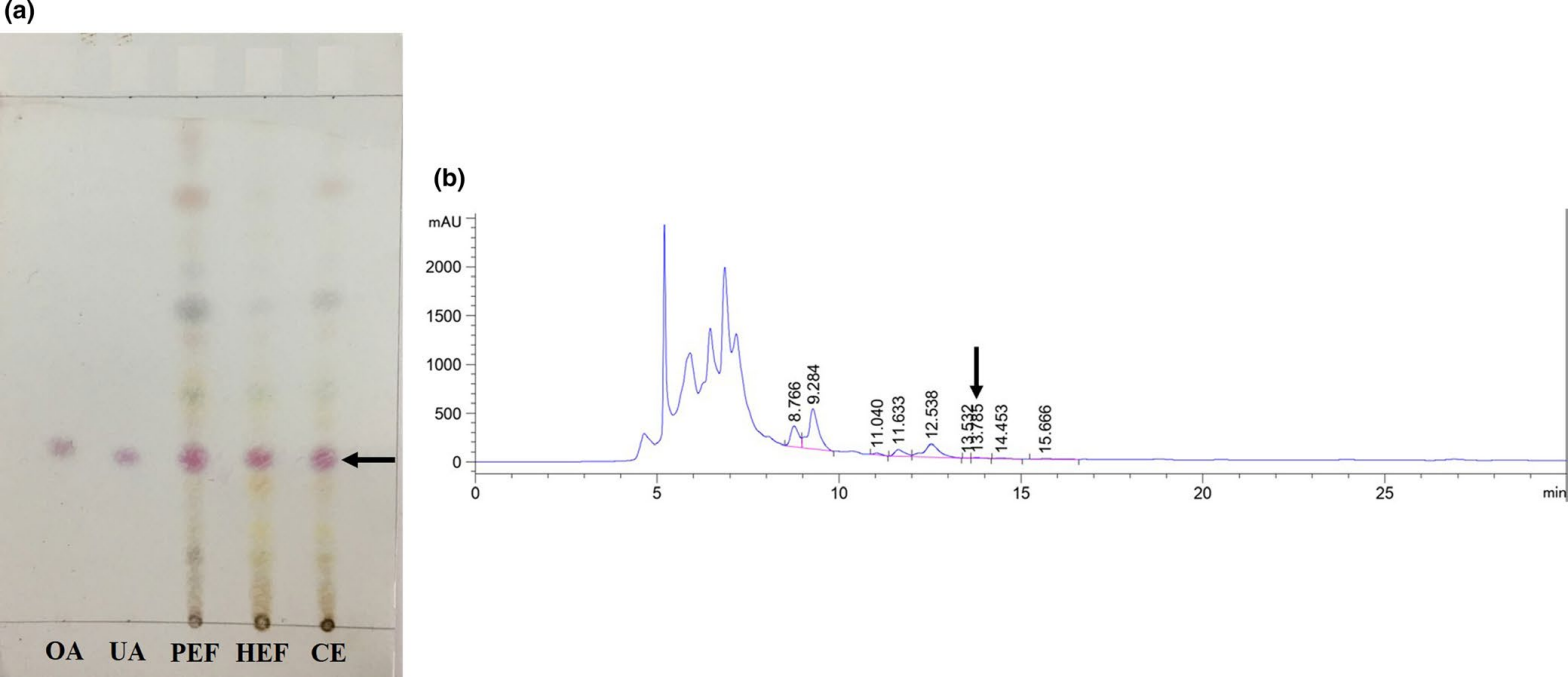
TLC (a) result of the *Severinia buxifolia* bark extracts (crude (CE), fraction petroleum ether (PEF) and hexane:ethyl acetate (HEF); OA: oleanolic acid; UA: ursolic acid) showing positive to ursolic acid (a triterpenoid component) and HPLC (b) chromatogram detected at 210 nm for the crude CE and the HEF fraction of *S. buxifolia* bark

We performed TLC and HPLC analyses as a part of the quality control of the *S. buxifolia* bark extracts. First, the TLC of crude extract and fractions was prepared using oleanolic acid and ursolic acid as markers of terpenoid compounds (Figure 6a). The retention factor (Rf) values of crude extract and fractions are shown in Table 1; Rf indicates the variability of the terpenoid content in *S. buxifolia* bark and all extracts containing ursolic acid. Conversely, oleanolic acid was not detected in all samples. The chromatogram revealed six, five, and four spots for crude CE, fractions PEF and HEF, respectively.

**TABLE 1:** Retention factor (Rf) value of TLC solvent system for the crude extract (CE) and the extract fractions (petroleum ether—PEF and hexane:ethyl acetate—HEF) of *Severinia buxifolia* bark

Extract name	Visualization	No. of spots	Rf value
Crude bark extract (CE)	Liebermann reagent	6	0.18 0.27 **0.31** 0.45 0.64 0.81
Petroleum ether bark extract fraction (PEF)	Liebermann reagent	5	0.12 **0.31** 0.42 0.59 080
Hexane:ethyl acetate bark extract fraction (HEF)	Liebermann reagent	4	0.13 0.26 **0.31** 043
Oleanolic acid	Liebermann reagent	1	0.33
Ursolic acid	Liebermann reagent	1	**0.31**

Note

Rf = retention factor; solvent system ‐ hexane:ethyl acetate: methanol; ratio: ‐ 8.2:1.8:0.5; v/v/v; Liebermann reagent: 10% H_2_SO_4_ in ethanol and 10% acetic anhydride.

Bold values in the Rf value means the Rf of spot has a value equal to Rf of standard (ursolic acid).

Based on the TLC levels, HPLC analyses were performed for all extracts, using ursolic acid as the reference standard. According to the results (Figure 6b), ursolic acid was found in all tested extracts of *S. buxifolia* bark. The ursolic acid value was obtained from the calibration curve *y* = 27.183*x* − 39.752, with *R* ^2^ = 0.9894 (Retention time (RT) = 13.78), where *x* is the absorbance unit and y is the peak area expressed as mAU.s. The contents of ursolic acid in the crude CE and the PEF and HEF fractions were 2.44 μg/g DW (peak area 26.55 mAU.s), 3.56 μg/g DW (peak area 68.66 mAUs), and 5.04 μg/g DW (peak area 97.21 mAU.s), respectively.

#### 3.2.4 In vitro anti‐inflammatory activity

##### Inhibition of albumin denaturation

Based on the results of the albumin denaturation assay, all extracts of *S. buxifolia* bark (crude CE and fractions PEF and HEF) effectively inhibited protein (albumin) denaturation caused by heat (Table 2). The maximum inhibition at a concentration of 200 μg/ml ranged from the crude extract to the PEF and HEF fractions, with inhibitions of 51.11%, 52.56%, and 69.58%, respectively, whereas aspirin produced a 82.04% inhibition. The inhibition of the albumin assay IC_50_ value significantly differed between the tested extracts (*p* < .001) (Table 3), ranging from 131.82 μg/ml for the HEF fraction to 197.42 μg/ml for the crude CE. The IC_50_ value of PEF (194.91 μg/ml) was not significantly lower than that of the crude CE. The IC_50_ value of aspirin was 58.96 μg/ml.

**TABLE 2:** In vitro anti‐inflammatory activity of the crude extract (CE) and the extract fractions (petrolium ether—PEF and hexane:ethyl acetate—HEF) of *Severinia buxifolia* bark

Activities	Concentration (μg/ml)	% Inhibition				
		CE	PEF	HEF	Aspirin	Indomethacin
Albumin denaturation	25	9.39^b^ ± 1.10	10.43^b^ ± 0.83	10.91^b^ ± 0.66	24.70^a^ ± 0.56	–
	50	18.24^b^ ± 1.13	18.99^b^ ± 2.86	20.97^b^ ± 1.77	43.78^a^ ± 1.07	–
	100	29.85^c^ ± 1.81	33.47^b^ ± 0.86	33.93^b^ ± 0.62	68.29^a^ ± 0.56	–
	200	51.11^c^ ± 0.63	52.56^c^ ± 2.21	69.58^b^ ± 0.97	82.04^a^ ± 0.46	–
Heat‐induced hemolysis	25	23.71^b^ ± 1.68	28.24^ab^ ± 3.17	31.34^a^ ± 2.49	33.48^a^ ± 2.49	–
	50	32.76^c^ ± 1.69	34.32^c^ ± 1.65	45.79^b^ ± 3.41	55.38^a^ ± 3.41	–
	100	42.80^c^ ± 1.74	46.37^c^ ± 0.77	52.53^b^ ± 2.07	67.88^a^ ± 2.07	–
	200	54.59^c^ ± 2.13	55.56^c^ ± 2.14	60.53^b^ ± 1.39	76.75^a^ ± 1.39	–
Proteinase inhibitory activity	25	14.44^b^ ± 6.01	21.25^ab^ ± 1.90	21.78^ab^ ± 4.11	24.64^a^ ± 1.14	–
	50	28.54^a^ ± 1.32	28.80^a^ ± 1.46	31.33^a^ ± 4.11	34.57^a^ ± 0.78	–
	100	43.28^c^ ± 1.74	45.61^bc^ ± 2.30	59.67^a^ ± 7.16	51.50^b^ ± 1.00	–
	200	55.27^c^ ± 2.04	57.93^c^ ± 1.91	66.50^b^ ± 086	78.45^a^ ± 3.00	–
Lipoxygenase inhibitory assay	25	27.84^c^ ± 0.76	30.36^bc^ ± 1.13	32.70^b^ ± 1.13	–	38.36^a^ ± 0.57
	50	38.67^c^ ± 1.12	42.34^b^ ± 0.76	44.78^ab^ ± 0.76	–	45.34^a^ ± 0.64
	100	49.99^c^ ± 1.61	53.75^b^ ± 0.52	56.29^b^ ± 0.52	–	65.31^a^ ± 0.91
	200	58.33^c^ ± 0.73	62.76^b^ ± 1.80	65.93^b^ ± 1.80	–	79.22^a^ ± 0.71

Note

All values are the mean ± *SD* (*n* = 3). Means within a line with different letters significantly differ by Tukey's test at *p* < .05.

Abbreviations: CE, crude bark extract; HEF, hexane:ethyl acetate bark extract fraction; PEF, petroleum ether bark extract fraction.

**TABLE 3:** IC_50_ value of in vitro anti‐inflammatory activity of the crude extract (CE) and the extract fractions (petrolium ether—PEF and hexane:ethyl acetate—HEF) of *Severinia buxifolia* bark

Extract name	IC_50_ values (μg/ml)			
	Albumin denaturation	Heat‐induced hemolysis	Proteinase inhibitory activity	Lipoxygenase inhibition assay
Crude CE	197.42^a^ ± 0.82	165.91^a^ ± 1.60	167.45^a^ ± 1.89	110.49^a^ ± 5.37
Fraction PEF	194.91^a^ ± 5.61	160.26^b^ ± 1.25	125.89^b^ ± 2.01	106.53^b^ ± 9.22
Fraction HEF	131.82^c^ ± 4.80	158.32^b^ ± 1.99	117.72^c^ ± 5.61	90.45^c^ ± 3.56
Aspirin	58.96^d^ ± 2.15	55.03^c^ ± 2.43	60.89^d^ ± 1.54	–
Indomethacin	–	–	–	52.25^d^ ± 3.56

Note

All values are the mean ± *SD* (*n* = 3). Means within a line with different letters significantly differ by Tukey's test at *p* < .05.

Abbreviations: crude CE, crude bark extract; fraction HEF, hexane:ethyl acetate bark extract fraction; fraction PEF, petroleum ether bark extract fraction.

##### Heat‐induced hemolysis

As shown in Table 2, the tested bark extracts inhibited heat‐induced lysis of the erythrocyte membrane in the range of 23.51%–60.53% at a concentration range of 25–200 μg/ml. The maximum inhibition of the crude CE and the fractions PEF and HEF was 200 μg/ml with 54.59%, 55.56%, and 60.53%, respectively. Aspirin demonstrated protection in the range of 33.48%–76.75%. The IC_50_ values significantly varied in the petroleum ether, hexane: ethyl acetate fractions, and the crude extract (*p* < .001), with 160.26, 158.32, and 165.91 μg/ml, respectively. The IC_50_ value of aspirin was 55.03 μg/ml (Table 3).

##### Proteinase inhibitory activity

The different *S. buxifolia* bark extracts (crude CE, fractions PEF and HEF) at various concentrations showed significant antiproteinase activity (*p* < .001) (Table 2). The maximum inhibition was 66.50% at 200 μg/ml of the HEF fraction, which was significantly higher than that of the PEF fraction (57.93%) and the crude CE (55.27%). Aspirin, the standard drug, showed the maximum inhibition of 80.11% at the same concentration (200 μg/ml). Table 3 shows that the IC_50_ values of petroleum ether and hexane: ethyl acetate fractions, as well as the crude extract, varied significantly (*p* = .008), with 125.89, 117.72, and 167.45 μg/ml, respectively. The IC_50_ value of aspirin was 60.89 μg/ml.

##### Lipoxygenase inhibition assay

The results of the in vitro LOX inhibitory assay of the bark extracts and the standard (indomethacin) are shown in Table 2. Inhibition increased according to the concentrations of the extracts, and all tested extracts had noticeable effects on the percentage inhibition of LOX. The maximum was 65.93% of the HEF fraction at 200 μg/ml. The inhibition of the PEF fraction (62.76%) was significantly higher than that of the crude extract (58.33%) at this concentration. The IC_50_ values of all tested extracts showed a significant difference (*p* < .001), with 106.53, 90.45, and 110.49 μg/ml, respectively. The IC_50_ value of the standard reference (indomethacin) was 52.25 μg/ml (Table 3).

## 4 DISCUSSION

The present study demonstrated that solvent–solvent fractionation of the *S. buxofolia* bark extracts can enhance the total terpenoid content as well as the anti‐inflammatory properties in vitro in some models, such as inhibition of protein (albumin) denaturation, heat‐induced hemolysis, proteinase activity, and LOX assay. In addition, the findings of some preliminary experiments on the effects of some factors of the maceration technique (solvent type, temperature, and time) on TTC and EY showed that hexane: acetone can be considered as the optimal solvent for extracting terpenoids from *S. buxifolia* bark at 46°C for 24 hr.

By applying the colorimetric assay with linalool as the standard reagent, the preliminary experiments of this study showed changes in the TTC of *S. buxifolia* extracts due to different maceration factors (solvent type, temperature, and time) (Figures 2 and 3). In all *S. buxifolia* extracts, the content of total terpenoids ranged from 453.70 to 842.59 mg linalool/g DW, and the yield varied from 2.40% to 5.10%. Single‐step extraction using hexane: acetone (1:1, v/v) was the optimal solvent for extracting the maximum amount of terpenoid components, and the extraction should be carried out over 24 hr at 46°C. The extraction technique is one of the most important steps to increase the contents of the desired bioactive compounds from plants (Azmir et al., 2013; Monton & Luprasong, 2019). One solvent is recommended as a good and optimal solvent for extraction based on its capacity in conserving the stability of the chemical structure of the targeted compounds (Do et al., 2014; Monton & Luprasong, 2019). For instance, the results of Thouri et al. (2017) revealed that when using Tunisian date seeds, the phenolic, flavonoid, and condensed tannin contents of the water and methanol extracts were higher than those of the acetone extracts. These authors showed that the polar solvent exhibited the highest amounts of bioactive compounds. In the present study, the TTC with regard to different solvents used for extractions from *S. buxifolia* bark showed the following order: hexane: acetone > acetone > hexane: ethyl acetate > hexane: diethyl ether > hexane. This result is in agreement with the observations of Harman‐Ware et al. (2016), who revealed that the optimal solvent for extracting the highest contents of terpenoids from pine lighter wood is a 1:1: hexane/acetone mixture. In addition to the extraction solvent, the temperature and time of maceration are also important parameters to be optimized, especially to minimize energy costs (Spigno et al., 2007). For example, the nitrate content of *Vernonia cinerea* varied according to the temperature (40–100°C) and time (10–60 min) of maceration (Monton & Luprasong, 2019). Lee et al. (2016) obtained an increase in the overall extract from agarwood leaves with an increase in operation temperature from 25 to 75°C. In the current study, we observed a significant variation of the TTC and EY of *S. buxifolia* extracts according to the temperature (6–86°C) and time (0–48 hr) of maceration, ranging from 453.70 to 842.59 μg/mL and 3.00% to 4.83%, respectively. Based on the TTC and EY levels as well as on the energy costs, we recommended a temperature of 46°C and a maceration time of 24 hr. Our results are in line with the findings of Bowman et al. (1997), who determined a maceration time of 24 hr for the isolation of terpenes from *Abies fraseri*.

Inflammation is a complex process in the human body and caused by physical injury and various chemicals (Chen et al., 2017; Manna & Jain, 2015; Parameswari et al., 2019). Nonsteroidal anti‐inflammatory drugs (NSAIDs) are most commonly used for the control of inflammatory conditions, based on the production of prostaglandin (Djuichou Nguemnang et al., 2019; Manna & Jain, 2015). However, they have various side effects, especially the formation of gastric ulcers caused by gastric irritation (Oguntibeju, 2018). In recent years, plant compounds have been considered as novel compounds with effective anti‐inflammatory activities (Parameswari et al., 2019; Shaikh et al., 2015; Upadhyay, 2020). Hoang et al. (2015) demonstrated that the presence of phenolics, flavonoids, alkaloids, and terpenoids in plant extracts may establish powerful anti‐inflammatory activity. In a previous study, it was reported that the various crude extracts of *S. buxifolia* branches (aqueous, methanol, ethanol, chloroform, dichloromethane, and acetone) are effective on the in vitro anti‐inflammatory activity (Truong et al., 2019). Anti‐inflammatory assays (i.e., inhibition of protein (albumin) denaturation, proteinase activity, heat‐induced hemolysis, and lipoxygenase (LOX) assay) of *S. buxifolia* were performed in this study, but with a focus on the fractionation of plant bark, which contains the terpenoid class as the main compound.

Protein denaturation leads to the destruction of the tertiary and secondary structure of proteins and is associated with the occurrence of inflammatory responses (Anoop & Bindu, 2015). Some anti‐inflammatory drugs, such as salicylic acid, phenylbutazone, and flufenamic acid, have a dose‐dependent ability to inhibit thermally induced protein denaturation (Djuichou Nguemnang et al., 2019). Albumin inhibition of *S. buxifolia* extracts ranged from 9.39% to 69.58%, from 25 to 200 μg/ml concentration of extracts (Table 2). The IC_50_ values of crude CE as well as PEF and HEF fractions of *S. buxifolia* bark were 197.41, 194.91, and 131.82 μg/ml, respectively (Table 3). The IC_50_ value of the standard reference, aspirin, was 58.96 μg/ml. Statistical analysis indicated there was a significant variation (*p* < .001) between means of different IC_50_ values of extracts. According to Osman et al. (2016), the capacity of the inhibition of protein denaturation from a substance can show the apparent potential for its anti‐inflammatory properties. Hence, the ability of the *S. buxifolia* bark extracts to carry out thermal denaturation of protein can provide evidence for their promising anti‐inflammatory activity. The ability of plant extracts to perform protein denaturation has been reported previously (Govindappa et al., 2011; Gunathilake et al., 2018; Osman et al., 2016) and might be due to the production of the lysosomal constituents of neutrophils at the inflammation site (Govindappa et al., 2011).

Cell vitality is a factor of membrane integrity, focusing on injuries of the RBCs' substances (Manoj et al., 2009). Chemical components with membrane‐stabilizing properties are expected to provide effective protection for cell membranes against injuries (Liu et al., 1992; Manoj et al., 2009; Shinde et al., 1999). Hence, we investigated membrane stabilization to further establish evidence for the anti‐inflammatory action of the plant components. The results showed that the crude extract and fractions have significant membrane‐stabilizing properties (Table 2). For heat‐induced hemolysis, the crude CE and the PEF and HEF fractions of *S. buxifolia* bark inhibited lysis of the erythrocyte membrane in the range of 23.71%–66.50% at a concentration range of 25–200 μg/ml. The protection of aspirin was demonstrated in the range of 33.48%–76.75%. The IC_50_ values varied significantly in the crude CE and the fractions of PEF and HEF (*p* < .001), with 165.91, 160.26, and 158.32 μg/ml, respectively. To the best of our knowledge, the mechanism for membrane stabilization of plant extracts/fractions has not yet been elucidated. According to some authors, extract/fractions liberate the membrane effect by increasing the cell surface area/volume ratio, leading to membrane expansion or cell shrinkage and interaction with membrane proteins (Chopade et al., 2012; Shinde et al., 1999). Thirumalaisamy et al. (2020) obtained a significant effect on the heat‐induced hemolysis of the methanolic extract and fractions (C and CS‐1 (identified as sitosterol)) of *Culcasia scandens*. The volatile oil of *Cedrus deodara* wood has a significant inhibition of heat‐induced hemolysis of erythrocytes in vitro (Shinde et al., 1999).

During the inflammatory responses, the leukocyte proteinases play an important role in the development of tissue damage (Gunathilake et al., 2018). In recent studies, many terpenoids contributed significantly to in vitro anti‐inflammatory activities of various plant extracts (Gallily et al., 2018; Marques et al., 2018; Namdar et al., 2019; Prakash, 2017). Thus, the presence of this component class in the plant extract maybe contributes to its anti‐inflammatory properties. Indeed, the results of the current study showed that the proteinase inhibition activity of *S. buxifolia* extracts increased according to the TTC content. The IC_50_ values of these extracts also significantly varied (*p* < .05) (Table 3). Recently, Thirumalaisamy et al. (2020) revealed the anti‐inflammatory efficiency of lupeol (a member of the triterpenoid class) from the chloroform extract fraction of *Crateva adansonii* leaves.

Lipoxygenases are one of the key enzymes in the biosynthesis of leukotrienes and play an important role in the response of some inflammatory diseases such as asthma, cancer, and arthritis (Chen et al., 2017; Gunathilake et al., 2018). In the anti‐inflammatory mechanism, arachidonic acid plays an important role in the responsive properties (Tallima & Ridi, 2017). According to Gardner (1991), the LOX pathway in plants is equivalent to the “arachidonic acid cascades” in animals. Thus, the inhibition of LOX in vitro can provide good evidence for the screening of plant extracts with anti‐inflammatory ability (Leelaprakash & Mohan, 2011). The observations of several previous studies showed that some medical plants have high LOX inhibitory activity (Gunathilake et al., 2018; Khasawneh et al., 2011; Leelaprakash & Mohan, 2011; Rackova et al., 2007). For example, *Gymnema lactiferum* extracts have a potentially high anti‐inflammatory effect based on the LOX assay (Gunathilake et al., 2018). According to Yoon and Baek (2005), plant polyphenols can block or interfere with the cascade process of arachidonic acid metabolism via LOX inhibitory activity. In the present study, the results of the LOX inhibitory differed significantly between the crude extract and the fractions (Table 2) (*p* < .001). Indomethacin, used as a standard, inhibited LOX in the range of 38.96%–79.22%. The IC_50_ values were also significantly different (Table 3) (*p* < .001). Generally, the LOX inhibition activity is proportional to the concentration of the crude extract and fractions used in the test. This is in line with the observations of Cipta Sari et al. (2017) who stated that the higher the LOX inhibitory assay, the higher the concentration of the *Garcinia* extract. Plant extracts containing phenolics, flavonoids, and terpenoids have involved to exhibit synergism during the inhibition of LOX activity (Bhat et al., 2019).

We used HPLC to determine the formed metabolites from the crude CE and the fractions PEF and HEF of *S. buxifolia* bark. Ursolic acid, a natural pentacyclic triterpenoid carboxylic acid, was identified in all samples, albeit with different contents: 2.44 μg/g DW of crude CE, 3.56 μg/g DW of fraction PEF, and 5.04 μg/g DW of fraction HEF. Compared to the inhibition of anti‐inflammatory activity in vitro, the ursolic acid content in the *S. buxifolia* bark extracts was proportional to the inhibition increase. Ursolic acid may exhibit potent anti‐inflammatory effects (Checker et al., 2012); these authors also recommended the application of ursolic acid in the treatment of inflammatory disorders. Ursolic acid is considered as the major bioactive component of several plant species and has a wide range of biological functions, such as anti‐inflammatory, anticancer, and antioxidative activities (Ikeda et al., 2008; Tsai & Yin, 2008). The anti‐inflammatory, anticarcinogenic, and proapoptotic activities of ursolic acid could be correlated to its potential to inhibit the immunoregulatory transcription factor NFkB in carcinogens and inflammatory responses (Checker et al., 2012; Shishodia et al., 2003).

## 5 CONCLUSIONS

From the *S. buxifolia* bark, the preliminary experiments of the present study were determined the optimal conditions of maceration technique (solvent type, temperature, and time) for extracting the terpenoid from *S. buxifolia* bark. The results showed that the mixture of hexane:acetone (1:1, v/v) was considered as the optimal solvent to obtain the *S. buxifolia* crude extract at 46°C for 24 hr of maceration. Different organic solvents like petroleum ether and mixture of hexane:ethyl acetate (85:15, v/v) were used to fractionate the phytochemical groups. Solvent fractionation was found to increase the TTC compared with crude extracts based on colorimetric assay using linalool as standard reagent. The samples were indicated to have in vitro anti‐inflammatory comparable with two reference standards (aspirin and indomethacin) based on the inhibition of albumin denaturation, proteinase activity, heat‐induced hemolysis, and lipoxygenase assay. The HEF fraction was mostly showed the highest in vitro anti‐inflammatory assays in comparison with the PEF fraction and the crude CE. In addition, one of composition of terpenoid, namely ursolic acid, was determined by HPLC method from the crude CE and the fractions PEF and HEF: 2.44 μg/g DW, 3.56 μg/g DW, and 5.04 μg/g DW, respectively. Further studies would be conducted in the next research to purify and isolate more components from fractions as well as crude extracts. Generally, this study investigated that solvent–solvent fractionation significantly influenced on TTC as well as in vitro anti‐inflammatory activity of plant extracts.

## CONFLICT OF INTEREST

The authors declare no financial or commercial conflict of interest. The authors confirm that this manuscript is an honest, accurate, and transparent account of the study being reported.

## Data Availability

The data used to support the findings of this study are available from the corresponding author upon request.
